# Adaptive changes to saccade amplitude and target localization do not require pre-saccadic target visibility

**DOI:** 10.1038/s41598-023-35434-8

**Published:** 2023-05-23

**Authors:** Frauke Heins, Jana Masselink, Joshua-Nikodemus Scherer, Markus Lappe

**Affiliations:** 1 Institute for Psychology, University of Münster, 48149 Münster, Germany; 2 Otto Creutzfeldt Center for Cognitive and Behavioral Neuroscience, University of Münster, 48149 Münster, Germany

**Keywords:** Motor control, Oculomotor system, Visual system

## Abstract

The accuracy of saccadic eye movements is maintained by saccadic adaptation, a learning mechanism that is proposed to rely on visual prediction error, i.e., a mismatch between the pre-saccadically predicted and post-saccadically experienced position of the saccade target. However, recent research indicates that saccadic adaptation might be driven by postdictive motor error, i.e., a retrospective estimation of the pre-saccadic target position based on the post-saccadic image. We investigated whether oculomotor behavior can be adapted based on post-saccadic target information alone. We measured eye movements and localization judgements as participants aimed saccades at an initially invisible target, which was always shown only after the saccade. Each such trial was followed by either a pre- or a post-saccadic localization trial. The target position was fixed for the first 100 trials of the experiment and, during the following 200 trials, successively shifted inward or outward. Saccade amplitude and the pre- and post-saccadic localization judgements adjusted to the changing target position. Our results suggest that post-saccadic information is sufficient to induce error-reducing adaptive changes in saccade amplitude and target localization, possibly reflecting continuous updating of the estimated pre-saccadic target location driven by postdictive motor error.

## Introduction

Saccadic eye movements are essential for clear vision of our environment as they align our fovea with objects of interest in the visual scene. Saccades remain accurate over the life span^1,2^, even in case of injury or disease^3–5^ although they are of such short durations that they cannot be guided by visual feedback online^6^. Instead, post-saccadic information is required for saccadic error evaluation^7–11^. If gaze repeatedly misses the target, a learning mechanism termed saccadic adaptation adapts the primary saccade to reduce the error. Such recalibration occurs due to physiological changes^3–5,12^, but it can also follow intra-saccadic manipulation of the visual scene^13^. In the double-step paradigm, the saccade target is repeatedly shifted while the eyes are in flight. Although this target step typically goes unnoticed^14–16^, the primary saccade amplitude gradually adjusts to the intra-saccadic manipulation^17^.

Saccadic adaptation also affects visuospatial perception. The apparent location of objects in the vicinity of the saccade target shifts in direction of the intra-saccadic target step, leading to mislocalization^18–22^. Mislocalization after saccade adaptation was observed when a briefly presented flash has to be localized (a) while staying fixated at the fixation point (i.e. in pre-saccadic space, referred to as pre-saccadic localization), and (b) after performing a saccade to the saccade target (i.e. requiring a spatial update of the flash position from pre- to post-saccadic space, referred to as post-saccadic localization). The magnitude of mislocalization and saccade adaptation at a specific position within the adaptation field is correlated^23,24^. This correlation between saccade targeting and the perceptual localization reflects the close connection between action and perception, which is consistently updated to ensure optimal perception for action. This process is often referred to as visuomotor learning.

It is a topic of ongoing debate which error signal drives visuomotor learning. Initially, the distance between the fovea and the post-saccadic target object, termed the visual error, was proposed. However, this assumption does not align with saccades being typically hypometric^6,25^ and not fully adjusting to the intra-saccadic target shift^9,10,17,26^. Those findings suggest that the learning process does not aim at minimizing visual error. Instead, it was proposed that a mismatch between the predicted and actual post-saccadic retinal target position drives visuomotor learning: visual prediction error^7,11,19,27^. The predicted post-saccadic target position is supposed to rely on an update of the pre-saccadic target position by the computed displacement of visual space, i.e. by an internal estimation of saccade size. This information is derived from a copy of the motor command, known as efference copy or corollary discharge^28–30^. However, there are three arguments against visual prediction error. (1) In previous oculomotor learning models the prediction usually went in as an assumption, e.g. the prediction is that the eye will always land on target or with a fixed target undershoot as measured in the baseline^31–33^. Recently, Masselink & Lappe^34^ measured the prediction during oculomotor learning based on a trans-saccadic localization approach. If visual prediction error is nullified, the prediction of the post-saccadic target position should match the actual position of the shifted post-saccadic target. This was not the case^34^. (2) Visual prediction error indicates a violation of visual stability which is inconsistent with saccadic suppression of displacement^14,15^. (3) Visual prediction error only optimizes visual prediction across a saccade, but not necessarily saccade performance. Particularly, visual prediction error is nullified when the post-saccadic target appears at the same retinal eccentricity as predicted, independently of how large this eccentricity is. This is why Masselink & Lappe^34^ proposed that oculomotor learning is guided by a postdictive motor error. In this model, the visuomotor system uses the post-saccadic target position to postdict where the target was located in pre-saccadic space, i.e. before saccade initiation, and then computes the saccade error with respect to this position. Learning from postdictive motor error (1) obtained a good model fit with the measured saccade and localization data, (2) is consistent with saccadic suppression of displacement, and (3) aims at optimizing saccade accuracy.

The current study examines whether saccade and target localization adjustments can be induced if only post-saccadic target information is available. In this case, a pre-saccadic target representation can only be built up postdictively, i.e. by backward modeling the target from post- to pre-saccadic coordinates, in order to guide saccade and localization adjustments. We conducted two experiments during which the target was presented only after the saccade.

Each experiment began with a phase in which the post-saccadic target was always presented in the same stable position. In this stable position phase, we studied how participants initially acquire information about the target, how they direct their saccades and how they localize targets before and after a saccade. We expect that, during the stable position phase, saccades and localization judgements should become more accurate and precise as repeated post-saccadic presentation of the target could be used to generate an increasingly veridical pre-saccadic target representation. After the stable position phase, the experiment continued with a displacement phase, in which the post-saccadic target was gradually presented further inward (Experiment 1) or outward (Experiment 2) across trials. We expect that, during the displacement phase, saccade endpoints and pre- and post-saccadic localization judgements follow the gradual target displacement, as the pre-saccadic representation of the target used for saccade targeting is updated after each saccade with respect to the post-saccadic error.

## Experiment 1: Inward target displacement

### Method

#### Sample

The sample consisted of 16 participants (8 female). The age of the participants ranged between 18 and 49 years (*M* = 27.00, *SD* = 7.21). All of them had normal or corrected-to-normal vision and gave written informed consent before participating in the study. The participants were compensated with either course credit or 8 €/h.

#### Experimental setup

The experiment was performed in a dark room below 0.01 cd/m^2^. All sources of light were eliminated. Subjects were seated with a distance of 62.5 cm in front of an Eizo FlexScan F930 monitor (Eizo, Hakusan, Japan). The monitor was running at a frame rate of 75 Hz with a screen resolution of 1152 × 870 pixels. The monitor and its edges were covered with a dark foil to prevent visibility of the monitor background light and to reduce the effects of phosphor persistence (afterglow). This eliminated visual references (e.g. screen borders) during the experiment^21,22,24,34^. Viewing was binocular and the right eye was recorded using the EyeLink 1000 (SR Research, Ontario, Canada) at a sampling frequency of 1000 Hz. The code for the experiment was written in MATLAB (R2018a; The MathWorks) and the Psychophysics Toolbox extension^35,36^ was used for stimulus presentation. A combined chin-forehead rest ensured a stable head position during the recording session. The experimental procedures followed the 2008 declaration of Helsinki and were approved by the ethics committee of the Department of Psychology and Sport Science of the University of Muenster.

#### Stimuli and procedure

**Figure 1:**
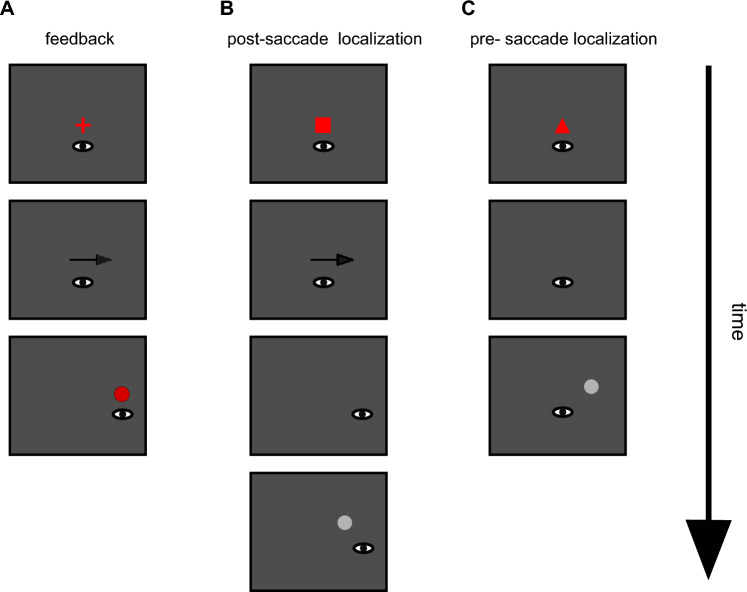
Sequence of events in the different trial types. (**A**) During a feedback trial, participants first looked at a fixation cross. Once it was removed, participants made a saccade to the position on the screen where they expected the target to be. When saccade onset was detected, the target appeared on the screen. (**B**) During a post-saccadic localization trial, participants first looked at a fixation square. Once the square was removed, they made a saccade to the position on the screen where they expected the target to be. After saccade landing, participants continued to fixate the landing position. 300 ms after saccade landing, the cursor appeared and participants indicated where they expected the target. (**C**) During a pre-saccadic localization trial, participants first looked at a fixation triangle and continued to hold fixation when the fixation marker was removed. 500 ms after the fixation had been removed, the cursor appeared and participants indicated where they expect the target.

The sequence of events in different trial types is depicted in Fig. 1. The experiment consisted of 300 trials in total. After every 100 trials the experiment paused to give the participants an opportunity to rest their eyes. The participants could self-initiate the next block by pressing the space bar. The trial sequence was as follows: feedback trial, post-saccadic localization trial, feedback trial, pre-saccadic localization trial, and so on. Thus, each feedback trial was always followed by either a pre- or post-saccadic localization. The target position in the feedback trials remained constant throughout the first 100 trials as the target always appeared 12° to the right of the fixation marker. The first 100 trials of the experiment are thus referred to as stable position phase. From then on, the target position was shifted 0.03° inward on each feedback trial. Thus, the final target position at the end of the experiment was 9° to the right of the fixation marker. The final 200 trials of the experiment are referred to as position displacement phase.

Before starting the main experiment, the participants performed practice trials until they were familiar with the tasks during the different types of trials. During the practice trials, the target always appeared at the same position and always to the left instead of the right of the fixation marker. This was done to avoid that participants would already begin to learn the target position before the start of the main experiment. Including instructions and practice trials, a recording session typically lasted just under an hour.

Stimuli were presented on a black background. Each trial began with the presentation of a red fixation marker 3° to the left of the screen center. The shape of the fixation marker was indicative of the trial type and the associated task. Participants had to fixate the fixation marker for a random time interval of 500 to 1000 ms until it was removed from the screen. If the eye left the fixation window (2.5 × 2.5°) around the fixation marker, a sinusoidal tone was played and the trial was aborted and restarted.

A fixation cross (0.5 × 0.5°) indicated a feedback trial (Fig. 1A). The removal of the fixation cross served as go-signal for the eye movement and participants had to make a saccade to the position on the screen where they expected the target to appear. Once saccade onset had been detected (i.e. the eye velocity exceeded 100°/s and the eye had travelled at least 3° toward the future target position), the target, a red circle (1° diameter) was drawn to the screen. It remained on screen for 500 to 1000 ms, providing post-saccadic feedback about the target position and thus the accuracy of the saccade. Then, the target was removed and the next trial started. Before the start of the main experiment, participants were told that the target would appear somewhere to the right of the fixation marker.

A fixation square (0.5 × 0.5°) indicated a post-saccadic localization trial (Fig. 1B). During post-saccadic localization trials, participants had to fixate the fixation square until it was removed. Upon removal of the fixation square, participants had to make a saccade to the position on the screen where the participants expected the target to appear. As soon as saccade landing was detected (eye velocity below 30°/s for three consecutive samples), participants had to hold fixation at the saccade landing position in total darkness. If their eye left the fixation window (3 × 3°) around the saccade landing position, a tone was played to remind the participant to hold a stable fixation at the saccade landing position. If more than three beeps occurred due to the eye leaving the post-saccadic fixation window, the trial was aborted and restarted. 300 ms after saccade landing was detected, a grey dot cursor (diameter 1°) was drawn to the screen. It appeared up to $\pm$ 3° to the left or right and up to $\pm$ 5° above or below the target position in the preceding feedback trial. The distribution of the cursor positions was centered around zero and counterbalanced to prevent any systematic effect of cursor position on the localization judgement. While the subjects held fixation at the saccade landing position, they moved the cursor toward the position where they expected the target using a multi-touch trackpad (Apple Inc, Cupertino, CA). After they had given their localization judgement, the screen turned black and the next trial began.

A fixation triangle (base = 0.5°, height = 0.5°) indicated a pre-saccadic localization trial (Fig. 1C). Participants had to fixate the triangle until it was removed from the screen. Then, they continued to fixate the position of the triangle in total darkness. If the eye left the fixation window (3 × 3° around the position of the fixation triangle), a tone was played to remind the participant to hold fixation. If more than one beep occurred, the trial was aborted and restarted. The cursor appeared 500 ms after the removal of the fixation triangle. Its position varied in the same manner around the target position as during the post-saccadic localization trials. While the subjects continued to fixate the position of the fixation marker in darkness, they moved the cursor to the position where they expected the target.

Any trial, regardless of the type of trial, could be repeated a maximum of twice. Otherwise it was skipped and the next trial began.

#### Data analysis

All trials with valid primary saccades (feedback trials), valid localization judgements (pre-saccadic localization trials) or both (post-saccadic localization trials) were included in the data analysis. Valid primary saccades were defined as having an amplitude between 4.5 and 18°, a peak velocity between 100 and 900°/s and a latency, measured as time between offset of the fixation marker and onset of the saccade, of at least 100 ms. Following these criteria, 86.03% of primary saccades, 88.92% of the post-saccadic localization judgements and 98.08% of the pre-saccadic localization judgements were included in the data analysis.

**Figure 2:**
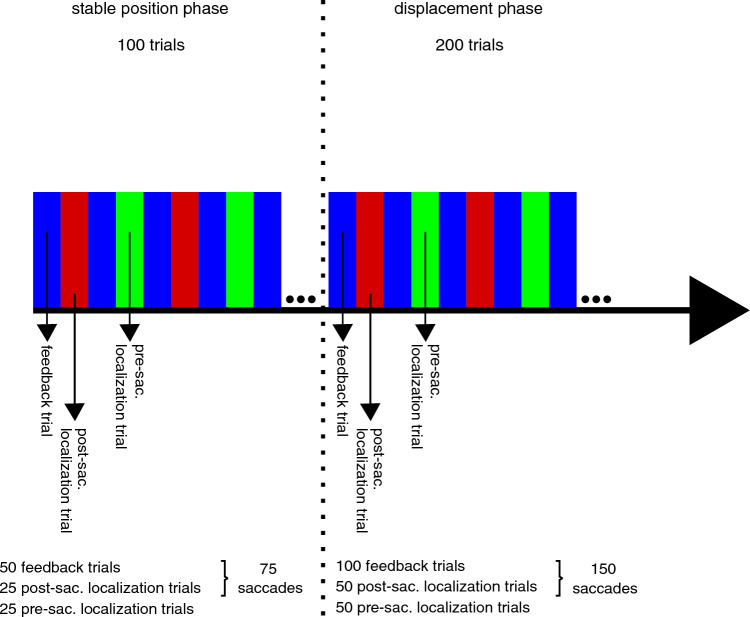
Order and number of trial types during the stable position phase (100 trials) and the displacement phase (200 trials). In each phase, feedback trials alternated with localization trials, which were either pre- or post-saccadic. For data analysis, saccades from the feedback trials and the post-saccadic localization trials were combined to yield 75 saccade trials overall in the stable position phase and 150 saccade trials overall in the displacement phase, and a total of 225 saccade trials over the entire experiment.

Saccade errors and localization errors were calculated to assess the accuracy of saccades and localization judgements. During feedback trials, saccade errors were calculated with respect to the position at which the target appeared following the saccade. During post-saccade localization trials, the saccade error was calculated with respect to the target position in the preceding feedback trial. In total participants made saccades in 225 trials (Fig. 2). The pre- and post-saccade localization errors were measured with respect to the target position in the preceding feedback trial. Throughout the experiment, 75 pre- and post-saccade localization judgements were made (Fig. 2).

We calculated the correlation between the saccade error in a given trial and the change in saccade amplitude from this trial to the upcoming trial. The Pearson correlation coefficient assumes independence of the individual data points, which does not apply to our case as we have multiple observations per participant. Instead of averaging the multiple observations for each participant to counteract the violation of the independence assumption, we used the repeated measures correlation available within the R-package “rmcorr” ^37^ that typically yields greater statistical power. The repeated measures correlation uses analysis of covariance to adjust for inter-individual variability, provides a linear fit for each individual participant using parallel regression lines with varying intercepts, and evaluates the overall within-subject association between two variables. Like the Pearson correlation coefficient, the repeated measures correlation ranges between − 1 and 1.

To assess if the saccade amplitude adjusts significantly to the continuous target displacement, the saccade gain was calculated. The saccade gain describes the ratio between the distance of the original target position to the fixation cross and the saccade amplitude. As the original target position was 12° to the right of the fixation cross, a gain of one indicates that the saccade had an amplitude of 12°. In Experiment 1, the final target position was 9° to the right of the fixation cross. A perfect adjustment to the gradual inward target shift therefore would be indicated by a saccade gain of 0.75. In Experiment 2, the final target position was 15° to the right of the fixation cross. A perfect adjustment to the gradual outward target shift would therefore be indicated by a saccade gain of 1.25. The same applies to pre- and post-saccadic localization gain.

We used the SMART method^38^ to assess saccade and localization errors as well as saccade and localization gain as a continuous time series throughout the experiment. Using the SMART method, one can compare the dependent variable over time against a baseline value and detect at which point in time the dependent variable differs significantly. The SMART method involves generating a smoothed time series of the data for each participant. We used a moving Gaussian window ($\sigma=$ 5 trials) for the temporal smoothing. Before the temporal smoothing was applied, saccade data from the trial types during which a saccade had to be made (feedback and post-saccade localization) trials was combined to a continuous time series. To do so, the dependent variable (saccade gain, saccade error) was matched with the independent variable (index of the saccade trial, ranging from 1 to 225). The same procedure was applied to the pre- and post-saccade localization data, and the localization data was matched with the index of the pre- or post-saccade localization trial (ranging from 1 to 75). Following the temporal smoothing, a weighted averaged time series is constructed across participants. This step ensures that participants with more data around a given time point (i.e. less missing data) contribute more to the group average than participants with more missing data around the respective time point. Subsequently, weighted t-tests for each time point of the time series are calculated. Because the dependent variable in a given trial is not independent of its value in a preceding or succeeding trial, clusters of temporally adjacent trials emerge. Significant clusters are defined as two or more consecutive time points for which the cluster strength exceeds a critical t-value. The cluster strength is defined as the sum of t-values for a cluster. For the permutation testing, condition labels are randomly shuffled for each permutation, and the sum of t-values for the largest cluster is added to the permutation distribution. The procedure is repeated 10.000 times. To identify significant clusters in the original data, the cluster strength is compared to the permutation distribution, and clusters within the original data with a cluster strength exceeding the 95^th^ percentile are considered significant. For significant clusters, the cluster strength (*t*), the critical t-value (*t*_*crit*_) and the corresponding *p*-value are reported.

The data preprocessing was done using MATLAB (R2018a) and the statistical analyses using R (version 4.1.2) and Python (version 3.9.12).

### Results

**Figure 3:**
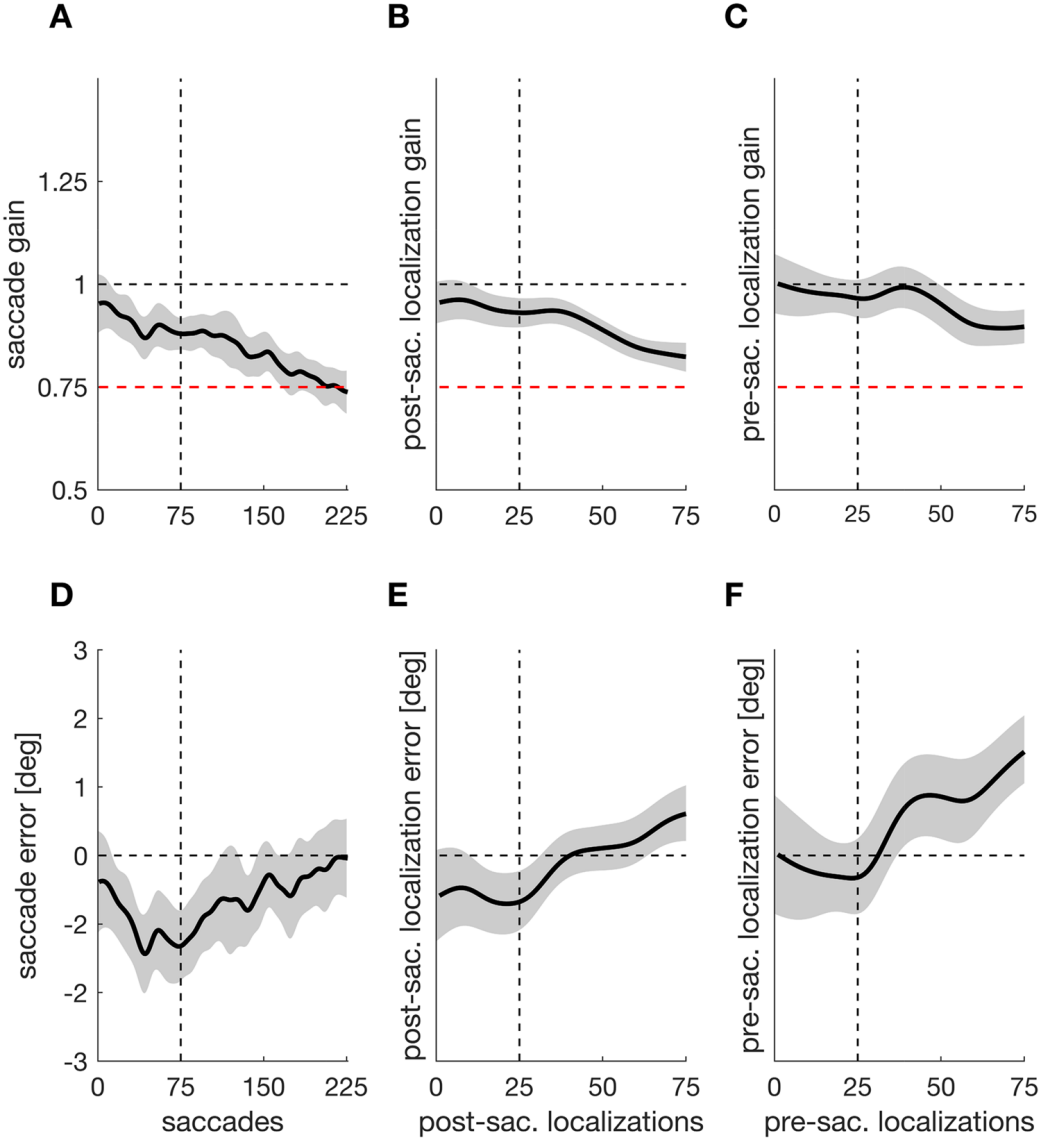
Saccade gain (**A**), post-saccadic localization gain (**B**) and pre-saccadic localization gain (**C**) as a function of trial number. The black horizontal dashed line indicates the baseline value of one, which corresponds to the target position during the stable position phase. The red horizontal line indicates a gain of 0.75, which corresponds to final position that the target reached at the end of the displacement phase. The vertical dashed line marks the transition from stable position to displacement period. Saccade error (**D**), post-saccadic localization error (**E**) and pre-saccadic localization error (**F**) as a function of trial. The horizontal dashed line indicates the baseline value of zero. Shaded areas indicate the 95% confidence interval.

We investigated if repeated presentation of a post-saccadic target alone is sufficient to develop a pre-saccadic representation of the target position that can guide saccade adjustments. If indeed a pre-saccadic target representation develops and determines the saccade vector, saccade amplitude and localizations should follow the gradual inward target displacement during the displacement phase. Thus, we assessed saccade and localization gain and smarted it against the baseline gain of one (Fig. 3A–C). The saccade gain was significantly below one from saccade trial 12 (experimental trial 15) onwards (*t*_*crit*_ = 51.412, *t* = 1051.00,* p* < 0.001), indicating that the saccade began to undershoot the target already during the baseline procedure. This might reflect the natural hypometric state of the saccade^6,25^. During the displacement phase, the saccade gain continuously decreased further and approached 0.75, reflecting optimal adjustment to the target shift (Fig. 3A). The post-saccade localization gain was significantly below one from post-saccade localization trial 12 (experimental trial 42) onwards (*t*_*crit*_ = 38.654, *t* = 478.604,* p* < 0.001), indicating that also the post-saccadic localization judgement undershot the original target position during the stable position phase. During the displacement phase, the gain continuously decreased, reflecting a partial adjustment to the target shift (Fig. 3B). The pre-saccadic localization gain was significantly below one from pre-saccade localization trial 50 (experimental trial 200) onwards (*t*_*crit*_ = 41.760, *t* = 116.930,* p* < 0.001). Thus, the pre-saccadic localization did not significantly undershoot the target position during the stable position phase, but began to gradually adjust to the target shift during the displacement phase (Fig. 3C).

If the repeated post-saccadic presentation of the target suffices to develop a postdicted target representation that guides saccade targeting, then saccade endpoints and localization judgements should become more accurate (i.e. land closer to the target position) during the baseline phase with stable target position. We thus assessed if saccade endpoints and localization judgements began to land closer to the target object during the baseline phase. We also evaluated the saccade and localization error during the displacement phase to see how they developed in response to the continuous target shifts. We smarted the errors against a baseline value of zero (Fig. 3D–F). The saccade error was significantly below zero between saccade trials 14 and 110, (experimental trials 17–146; *t*_*crit*_ = 53.3119, *t* = 406.158,* p* < 0.001). Thus, the saccade was not accurate at the end of the stable position phase. During the displacement phase, however, the saccade error approached zero until it was no longer significant, indicating that the saccade landed more accurately on target (Fig. 3D). The post-saccadic localization error was also significantly below zero between post-saccade localization trials 11 and 31 (experimental trials 44–122; *t*_*crit*_ = 38.407, *t* = 62.722,* p* = 0.007). Thus, the post-saccadic localization judgement was not accurate by the end of the stable position phase. During the displacement phase, the post-saccadic localization error approached zero until it was no longer significant from post-saccadic localization trial 31 onwards until the end of the displacement phase (Fig. 3E), consistent with the finding that the saccade landed more accurately on target.

The pre-saccadic localization error was above zero from pre-saccade localization trial 37 (experimental trial 148) onwards (*t*_*crit*_ = 41.144, *t* = 148.091, *p* < 0.001). It did not deviate significantly from zero throughout the stable position phase but increasingly overshot the saccade target position throughout the displacement phase (Fig. 3F).

**Figure 4:**
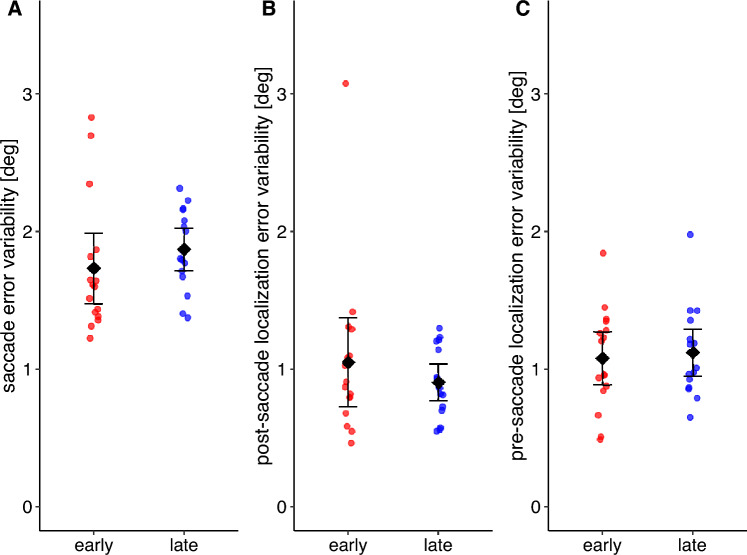
Mean intraindividual variability of saccade error (**A**), post-saccadic localization error (**B**) and pre-saccadic localization error (**C**) during the early (red) and late (blue) stable position phase. The error bars indicate the 95% confidence interval.

If the motor error is postdicted and used to develop a pre-saccadic target representation, then saccades and localization judgements could also become more precise (i.e. less variable). We thus assessed if the intraindividual variation of saccade as well as pre- and post-saccadic localization errors decreased throughout the stable position phase. For each participant, we calculated the standard deviation of the saccade error during the first and last 25% of the trials during the stable position phase (19 trials). The same applies to the pre- and post-saccade localization judgements (7 trials). Saccade error variability did not decrease from early (*M* = 1.73°, *SD* = 0.48°) to late stable position phase (*M* = 1.87°, *SD* = 0.29°; *t*(15) = − 1.089, *p* = 0.853, one-sided t-test; Fig. 4A). The same applies to the post-saccadic localization error variability (early: *M* = 1.05°, *SD* = 0.61, late: *M* = 0.90°, *SD* = 0.25°;* p* = 0.248, one-sided Wilcoxon signed rank test; Fig. 4B) and the pre-saccadic localization error variability (early: *M* = 1.08°, *SD* = 0.36°, late: *M* = 1.12°, *SD* = 0.32°; *t*(15) = −0.363, *p* = 0.639, one-sided t-test; Fig. 4C).

**Figure 5:**
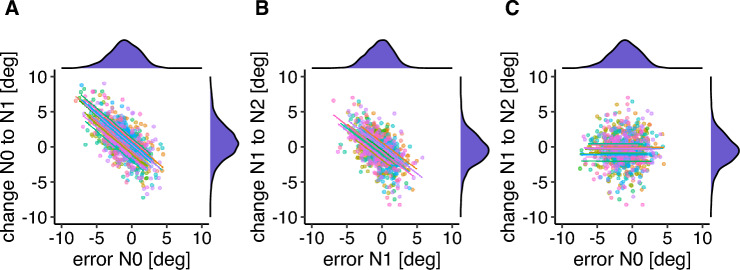
Correlation between the saccade error in a feedback trial and the change in saccade amplitude from this trial to the following no feedback post-saccade localization trial (**A**), between the saccade error in a no feedback post-saccade localization trial and the change in saccade amplitude from this trial to the next feedback trial (**B**) and between the saccade error in a feedback trial and the change in saccade amplitude from the following no feedback post-saccade localization trial to the next feedback trial (**C**). Each color marks one individual participant. Density plots for the saccade error and the saccade change are shown on the corresponding axes.

If the saccade motor error is postdicted from the post-saccadic image and used to update the estimated pre-saccadic target location, one would expect a strong correlation between the saccade error in a feedback trial (N0) and the change in saccade amplitude in the following post-saccade localization trial (N1). This correlation between the saccade error in N0 and the change in saccade amplitude from N0 to N1 was significant (*r* = − 0.717, *p* < 0.001, *CI* = [− 0.747, − 0.680]), indicating that if the saccade error was positive (i.e. the saccade overshot the target in a feedback trial), the saccade amplitude decreased in the following post-saccade localization trial (Fig. 5A). Further, we postulated that a representation of the saccade target object is generated from the repeated presentation of post-saccadic feedback. We expected that, in the absence of a visible saccade target, this target representation would be used for saccade targeting. This target representation could also be used for post-saccadic error estimation and the corresponding adjustment of saccade amplitude in the absence of visual targeting information. If this were true, then the saccade error in a post-saccade localization trial (N1) and the saccade change from N1 to N2 (the following feedback trial) should be related. The significant correlation between the saccade error in N1 and the saccade change from N1 to N2 (*r*− 0.559, *p* < 0.001, *CI* = [− 0.602 − 0.51]; Fig. 5B) confirmed that the internal target representation also guides the error evaluation following a saccade. If the error evaluation following a saccade in post-saccade localization trials relies on a comparison between the saccade landing position and the internal representation of the target location, then the saccade error in a feedback trial (N0) should not correlate with the change in saccade amplitude from N1 (following post-saccade localization trial) to N2 (following feedback trial). The non-significant correlation between the saccade error in feedback trial N0 and the amplitude change from N1 to N2 (*r* = − 0.002, *p* = 0.957, *CI* = [− 0.071, 0.060]; Fig. 5C) confirms that the amplitude change does not rely on the last available visible post-saccadic feedback. Note that, as the confidence intervals do not overlap, the correlation between the saccade error in a feedback trial and the following amplitude change was stronger than the correlation between the saccade error in a post-saccade localization trial and the following amplitude change. Thus, the relation between an error estimated with respect to a visual post-saccadic target and the following amplitude change is significantly stronger than the relation between an error estimated with respect to a representation of the target position and the following amplitude change.

Saccade latency was calculated relative to fixation cross offset. Saccade latency was not different between feedback (*M* = 504.41 ms, *SD* = 275.71 ms) and post-saccade localization trials (*M* = 500.66 ms, *SD* = 189.74 ms; *p* = 0.597, two-sided Wilcoxon signed rank test). Further, saccade latency did not differ significantly between stable position (*M* = 524.58 ms, *SD* = 226.74 ms) and displacement phase (*M* = 486.89 ms, *SD* = 232.40 ms; *t*(15), *p* = 0.112, two-sided t-test).

## Experiment 2: Outward target displacement

In Experiment 1, the saccade and localization gain adapted to the gradual inward shift of the target position during the displacement phase, indicating that visuomotor learning can occur even if the target is presented only after the eye movement. Analysis of the saccade error in the stable position and displacement phase, i.e. the accuracy of the saccade, showed a more complex picture. Over the course of the stable position period of Experiment 1, an undershoot of the target position developed. This undershoot might reflect the establishment of the natural hypometria of saccades^6,25^. Afterwards, during the learning phase, the saccades became increasingly accurate until the saccade error reached zero. This could either suggest that there was continuous learning during the displacement phase that optimized saccade accuracy, or it could be a byproduct of the inward target shift, which moved the target closer to the hypometric saccade endpoint established in the stable position phase. In Experiment 2, we aimed to investigate if the effects found for inward target displacement would also occur following outward target displacement, and more specifically, we aimed at assessing if the saccades would also become accurate when target displacement was not in direction of the hypometria.

### Method

#### Sample

The sample consisted of 18 participants (9 female). Their age ranged between 18 and 49 years (*M* = 26.61, *SD* = 7.31). All participants had normal or corrected-to-normal vision. They gave written informed consent before participating in the study. The participants were compensated with either course credit or 8 €/h. Two participants were not included in the data analysis. In one case, the inclusion criteria for the primary saccades were violated in more than 50% of the trials. In the second case, we did not receive any data due to a technical issue.

#### Experimental setup, stimuli and procedure

The overall procedure was the same as in Experiment 1. The only difference was the direction of the target displacement throughout the displacement period. Instead of shifting the target toward the fixation marker, the target position shifted 0.03° in outward direction each feedback trial, resulting in a final target position at 15° to the right of the fixation marker.

#### Data analysis

The same inclusion criteria as in Experiment 1 were applied to primary saccades and localization judgements. Following this, 84.64% of the primary saccades, 87.50% of the post-saccadic localization judgements and 99.67% of the pre-saccadic localization judgements were included in the data analysis which otherwise corresponds to the analysis following Experiment 1.

### Results

**Figure 6:**
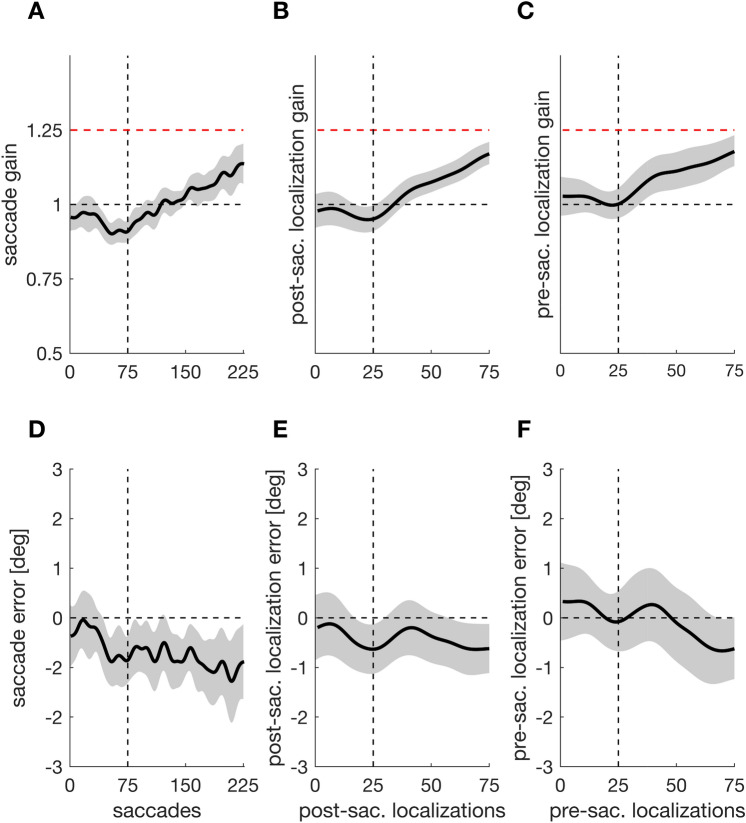
Saccade gain (**A**), post-saccadic localization gain (**B**) and pre-saccadic localization gain (**C**) as a function of trial. The black horizontal dashed line indicates the baseline value of one, which corresponds to the target position during the stable position phase. The red horizontal line indicates a gain of 1.25, which corresponds to final position that the target reached at the end of the displacement phase. The vertical dashed line marks the transition from stable position to displacement period. Saccade error (**D**), post-saccadic localization error (**E**) and pre-saccadic localization error (**F**) as a function of trial. The horizontal dashed line indicates the baseline value of zero. Shaded areas indicate the 95% confidence interval.

In Experiment 2, we expected saccade amplitude and localizations to follow the gradual target displacement in outward direction. Thus, we assessed the saccade and localization gain. The saccade gain was significantly smaller than one between saccade trials 43 and 117 (experimental trials 57 to 155; *t*_*crit*_ = 52.298, *t* = 240.094,* p* < 0.001; Fig. 6A) because the saccade undershot the target during the stable position phase. From saccade trial 125 (experimental trial 166) onwards, the saccade gain was significantly greater than one (*t*_*crit*_ = 52.298, *t* = 188.183,* p* < 0.001; Fig. 6A) as the saccade amplitude gradually increased throughout the displacement phase. The post-saccade localization gain was significantly greater than one from post-saccade localization trial 39 (experimental trial 154) onwards (*t*_*crit*_ = 40.053, *t* = 215.492,* p* < 0.001; Fig. 6B) as the localization judgement gradually shifted in direction of the target displacement. The pre-saccadic localization gain exceeded one from pre-saccade localization trial 34 (experimental trial 136) onwards (*t*_*crit*_ = 39.736, *t* = 190.042,* p* < 0.001; Fig. 6C) as the pre-saccadic localization judgement also shifted in outward direction.

We also assessed the accuracy of saccade endpoints and localization judgements over time. The saccade errors were significantly below zero between saccade trials 43 and 117, corresponding to experimental trials 57 to 155 (*t*_*crit*_ = 52.050, *t* = 219.037,* p* < 0.001), and from saccade trial 125 (experimental trial 166) onwards (*t*_*crit*_ = 52.050, *t* = 325.669,* p* < 0.001; Fig. 6D). Thus, the saccade hypometria was established during the stable position phase, as in Experiment 1. Remarkably, the saccade undershot the target position also during the displacement procedure. This is further evidence that the saccade control in our experiments establishes the natural hypometric state of saccadic eye movements^6,25^. The post-saccade localization errors were significantly below zero from post-saccade localization trial 58 (experimental trial 230) onwards (*t*_*crit*_ = 39.769, *t* = 45.558,* p* < 0.032; Fig. 6E), indicating that during the displacement procedure, participants began to localize the target inward from its actual position. The pre-saccadic localization error did not deviate from zero throughout the entire experiment (Fig. 6F).

**Figure 7:**
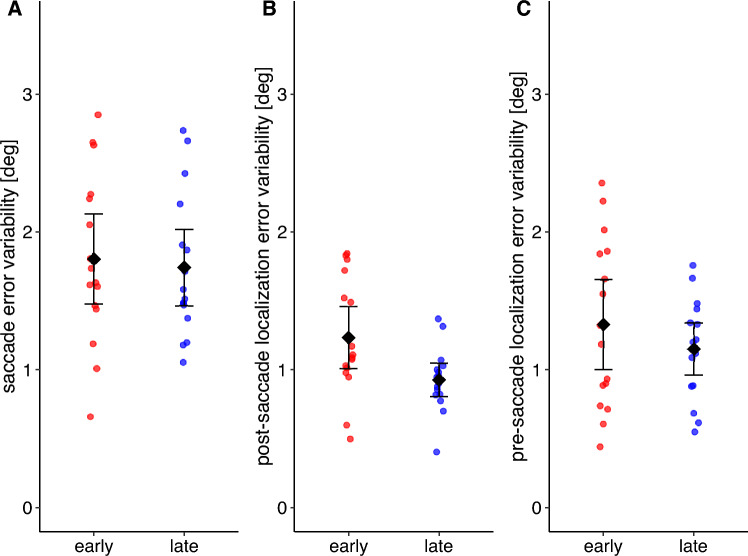
Mean intraindividual variability of saccade error (**A**), post-saccadic localization error (**B**) and pre-saccadic localization error (**C**) during the early (red) and late (blue) stable position phase. The error bars indicate the 95% confidence interval.

Saccade and localization precision were compared between early and late stable position phase. Saccade error variability did not decrease from early (*M* = 1.80°, *SD* = 0.61°) to late stable position phase (*M* = 1.74°, *SD* = 0.52°; *t*(15) = 0.372, *p* = 0.358, one-sided paired t-test; Fig. 7A). The variability of the post-saccadic localization error decreased from early (*M* = 1.23°, *SD* = 0.42°) to late stable position phase (*M* = 0.93°, *SD* = 0.23°; *t*(15) = 2.927, *p* = 0.005, one-sided paired t-test; Fig. 7B). The pre-saccadic localization error variability did not decrease from early (*M* = 1.32°, *SD* = 0.61°) to late stable position phase (*M* = 1.15°, *SD* = 0.36°; *t*(15) = 0.921, *p* = 0.186, one-sided paired t-test; Fig. 7C). Thus, precision increased only for post-saccade localization judgements.

**Figure 8:**
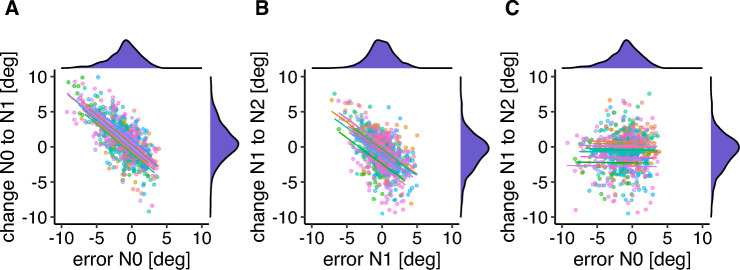
Correlation between the saccade error in a feedback trial and the change in saccade amplitude from this trial to the following no feedback post-saccade localization trial (**A**), between the saccade error in a no feedback post-saccade localization trial and the change in saccade amplitude from this trial to the next feedback trial (**B**) and between the saccade error in a feedback trial and the change in saccade amplitude from the following no feedback post-saccade localization trial to the next feedback trial (**C**). Each color marks one individual participant. Density plots for the saccade error and the saccade change are shown on the corresponding axes.

The correlation between the saccade error in a feedback trial (N0) and the change from saccade amplitude from this feedback trial to the subsequent post-saccade localization trial (N1) was significant (*r* = − 0.674, *p* < 0.001, *CI* = [− 0.709 − 0.636]; Fig. 8A), indicating that the error signal led to an adjustment of saccade amplitude in the upcoming trial. The correlation between the saccade error in a post-saccade localization trial (N1) and the amplitude change from this trial to the following feedback trial (N2) was significant (*r* = − 0.536,* p* < 0.001, *CI* = [− 0.581 − 0.487]; Fig. 8B), indicating the representation of the target position can also evoke adaptive changes to saccade amplitude. As in Experiment 1, the relation between an error estimated with respect to a visual post-saccadic target and the following amplitude change was stronger than the relation between an error estimated with respect to a representation of the target position and the following amplitude change. The non-significant correlation between the saccade error in a feedback trial (N0) and the amplitude change from the following post-saccade localization trial (N1) to the next feedback trial (N2) confirms that the amplitude change does not rely on the last available visible post-saccadic feedback (*r* = − 0.011, *p* = 0.768, *CI* = [− 0.082 0.061]; Fig. 8C).

Saccade latency (relative to fixation cross offset) was not different between feedback (*M* = 524.77 ms, *SD* = 326.36 ms) and post-saccade localization trials (*M* = 562.45 ms, *SD* = 329.87 ms; *t*(15) = -1.947, *p* = 0.070, two-sided t-test) but decreased between stable position (*M* = 619.66 ms, *SD* = 384.36 ms) and displacement phases (*M* = 495.98 ms, *SD* = 301.40 ms; *p* < 0.001, two-sided Wilcoxon signed rank test). This decrease in latency might reflect that participants, throughout the initial stable position phase, become familiar with the different trial types and their associated tasks and therefore need less preparation time before initiating eye movements.

## Discussion

We investigated visuomotor learning using a novel paradigm in which the target is presented only after the eye movement. We found that saccade amplitudes and localization judgements were adjusted to the gradual shift in target position during the displacement phase, both in inward and outward direction. This finding is in line with our first hypothesis. We expected that, in the absence of a visual pre-saccadic target object, a representation of the pre-saccadic target location would be generated based on a postdictive process. This process is supposed to integrate the post-saccadic target position with the computed displacement of visual space (i.e. the internally estimated saccade size obtained from efference copy information) to compute where the pre-saccadic target was. Because no visual target was available before the eye movement, only the postdicted pre-saccadic target representation could guide saccade targeting. We expected that the gradual target shift during the learning phase would lead to a post-saccadic error, which in turn would lead to an update of the pre-saccadic estimate of the target location and associated changes in saccade amplitude and localization judgements. Saccade as well as pre- and post-saccade localization gain decreased significantly following inward target displacement and increased significantly following outward target displacement. Thus, the pre-saccadic target representation was indeed updated in response to the post-saccadic error.

Our finding is in alignment with the model of Masselink & Lappe^34^, who suggested that visuomotor learning relies on postdictive motor error, i.e. a postdictive update of space after saccade landing. According to their framework, the internal representation of the pre-saccadic target location is postdicted from post-saccadic visual information using the computed displacement of visual space caused by the saccade. In case of a postdictive motor error, the world is assumed stable and the error is attributed internally. To reduce the error, the post-saccadic target location is postdicted into pre-saccadic coordinates and the representation of the pre-saccadic target location is updated accordingly. This postdicted pre-saccadic target representation drives error reducing adaptive changes to space perception and saccade targeting. In our current study, saccade targeting and localizing relied inevitably on the postdicted pre-saccadic target representation only. This target representation had to be constructed from post-saccadic input as no pre-saccadic target was ever presented.

The correlation analysis confirms that the post-saccadic error in a trial with post-saccadic target information influences the saccade amplitude in the immediately following trial, i.e. it suggests that the post-saccadic error calculated from the post-saccadic visual image was used to correct saccade targeting in the next trial. Moreover, the correlation between the saccade error in a post-saccade localization trial and the change in saccade amplitude in the immediately following trial provides an additional interesting insight. It suggests that even though no target information was available after the saccade, participants still estimated a saccade error and adjusted their future saccade amplitude accordingly. To do this, participants may have used their internal representation of the target location, which they had developed over time from the feedback trials. This suggests that the learned target representation was also used to evaluate the saccade error in the absence of post-saccadic target information.

We also assessed the accuracy of saccades and localization judgements. We expected that, because of repeated post-saccadic target presentation, an increasingly veridical representation of the target position would develop during the initial phase with stable position and lead to increasingly accurate saccades and localization judgements. Contrary to our expectation, the saccade endpoint did not adjust completely to the target during the stable position phase, neither in Experiment 1 nor in Experiment 2. In both experiments, the saccade endpoint significantly undershot the saccade target position by the end of the stable position period. This saccade error might have developed because the pre-saccadic target representation used for saccade targeting did not get more accurate over time, or it might reflect the natural tendency of saccades to fall short of the target^6,25^. The development of the saccade error in the displacement phase allowed to distinguish between these possibilities. Following inward target shifts, the saccade error approached zero and saccades became accurate. However, following outward target shifts, the saccade error remained significant during the displacement period as the saccade continued to undershoot the target. Together, these findings suggest that the target representation was learned successfully and the error arose from the natural hypometry of saccades. Thus, the full reduction of the saccade endpoint error during the displacement period of Experiment 1 might have occurred because the inward shift of the target counteracted the natural hypometric state of saccades. This would account for the difference in saccade error following inward and outward target displacement.

The development of post-saccadic localization judgements closely followed that of the saccade endpoints. Saccade gain and post-saccade localization gain developed in parallel following both inward and outward target displacement. However, the magnitude of adaptation to the target displacement was not necessarily equal. Following inward target shifts, the post-saccade localization judgements began to undershoot the target, similar to the saccade endpoints. Following outward target shifts, the post-saccade localization errors did not deviate significantly from zero during the entire baseline phase. During the displacement phase, post-saccade localization judgements following inward target shifts became more accurate over time. Following outward target shifts, the post-saccade localization judgements did not become more accurate. Thus, the pattern of results is similar for saccade endpoints and post-saccade localization judgements following outward target steps (hypometric during displacement) and following inward target steps (hypometric during stable position, accurate during displacement), indicating that the expected target position was similar before and after the saccade. The results suggest that the computed displacement of visual space, which is based on efference copy information and must be used for post-saccade localization, adapts in parallel with the saccade amplitude, but not necessarily with the exact same gain, which accounts for possible differences in the error magnitude for saccades and post-saccadic localizations.

The pre-saccadic localization judgements were accurate during the stable position phase of both Experiment 1 and 2. During the displacement phase, they became less accurate following inward target shifts and remained accurate following outward target shifts. This difference might have developed due to stronger alterations to the pre-saccadic representation of the target position following outward than following inward target displacement. For saccade adaptation, it has been suggested that the increase in saccade amplitude following intra-saccadic outward target displacement relies more on target remapping while the decrease of saccade amplitude following inward target displacement is attributed to internal adjustment of the ongoing motor performance^22,39–43^. Possibly, even though the target was not displaced during the saccade in the current study and we did not study conventional saccade adaptation, the mismatch between pre-saccadic representation of the target position, the post-saccadic visual image and the computed displacement of visual space might still have induced an update of the representation of the target position that was more pronounced following outward than inward target displacement. Further, the pre-saccadic localization judgement might reflect the true updated target position rather than the post-saccadic localization judgement because it is not affected by an executed saccade^43^.

Contrary to our second expectation, we found no evidence for an increase in precision of saccades or localization judgements during the stable position phase. The only significant increase in precision occurred for the post-saccadic localization judgement in Experiment 2. Thus, the results do not prove that the target representation became more reliable over time and thus caused less variable saccade endpoints or localization judgements. However, it is possible that the target representation developed too quickly to be captured by a comparison between early and late stable position or displacement phase. Indeed, to measure variability we used the first 25% of trials. If learning was rapid, then accuracy might have decreased already in the first few trials and precision would be stable from thereon.

The concept of building a postdictive pre-saccadic target representation that we propose here is closely related to building a spatiotopic map, i.e. a representation of the world in external coordinates. Saccades and spatiotopic representations exhibit a circular relationship. Saccadic eye movements serve to build spatiotopic representations by adding an additional visual snapshot with every saccade, and spatiotopic representations serve to plan saccades by representing the locations of objects in external space^44–46^. How might a visual snapshot following the completion of a saccade be attached to a spatiotopic map? The post-saccadic snapshot has to be spatially related to the pre-saccadic snapshot on the spatiotopic map. Like for trans-saccadic prediction, which performs a transformation from pre- to post-saccadic space, a relation from post-saccadic to pre-saccadic view can be established by an internal estimation about the size of the saccade, i.e. the computed displacement of visual space based on efference copy information. This transformation process from post- to pre-saccadic coordinates, no matter whether it is performed on a retinal or on a spatiotopic map, is postdiction. In our paradigm, the pre-saccadic target was not visible such that before saccade initiation, it could not be predicted to the post-saccadic scene. However, after saccade completion, the visible post-saccadic target could be postdicted to the pre-saccadic visual scene to learn from error and adjust saccade targeting.

In the conventional saccadic adaptation paradigm, the visual target is initially available to plan the saccade^13,17^ and to predict where the target will be in the visual field after the saccade^47,48^. The mismatch between this predicted position and the actual post-saccadic retinal position of the target is the visual prediction error. Several prior models proposed that saccadic adaptation is driven by the aim to nullify the visual prediction error^7,11,19,27^. Masselink & Lappe^34^ recently showed that this is not the case. They measured, for the first time, the prediction during saccadic adaptation by comparing pre- and post-saccadic localization. They found that prediction error minimization could not explain the adaptation since the prediction of the post-saccadic target position did not match the actual position of the shifted post-saccadic target after learning had converged. For example, after learning from outward target steps, the shifted post-saccadic target appeared outward of the saccade landing location while the target was predicted to appear inward of the saccade landing location. Instead, Masselink and Lappe proposed that oculomotor learning is guided by the postdictive motor error by which the visuomotor system uses the post-saccadic target position combined with an internal estimate of saccade size to postdict where the target was located in pre-saccadic space, i.e. before saccade initiation, and then computes the saccade motor error with respect to this position. They argued that learning from postdictive motor error, unlike learning from visual prediction error, provided a good model fit to the saccade and localization data, is consistent with saccadic suppression of displacement, and aims at optimizing saccade accuracy (instead of optimizing visual predictions). The present data adds that postdictive processes can also be used to construct a target representation that is never visually present before the saccade, and that this target representation can serve as a functional substitute for a visible pre-saccadic target, which in conventional saccadic adaptation instructs the saccade motor command.

Although learning from postdictive motor error does not involve a construction of visual prediction error it does involve similar predictive processes. Like models of visual prediction error, the postdictive motor error model relies on an estimation of the saccade’s effect on spatial representation, a computation of the displacement of visual space caused by the saccade based on an internal estimation of saccade size, or efference copy signal. The difference between the two models lies in how this prediction is used. In postdictive motor learning the computed displacement of visual space is used to postdictively estimate the location that the motor command should optimally have been targeted rather than predict where the target should have been after the saccade.

In summary, we observed that saccade amplitude and localization judgements adjusted to a gradually changing target position in a novel visuomotor learning paradigm that requires participants to perform saccades without a visible pre-saccadic target. The error-reducing changes that followed both inward and outward target shifts resemble the adaptive changes following intra-saccadic target displacement. However, possible differences between visuomotor learning following post-saccadic target presentation only and conventional saccade adaptation should be further investigated. It might prove interesting to study if visuomotor learning following post-saccadic target presentation only evokes similar aftereffects like saccadic adaptation, or if the neural recalibration following saccade adaptation requires the presence of a pre-saccadic target.

Taken together, our results suggest that oculomotor learning is possible from post-saccadic visual input alone. Both saccades and localization judgements changed in accordance with the shifting target position, indicating that no comparison between a physical pre- and post-saccadic target was required to induce adaptive, error reducing changes. It seems that our participants used the available post-saccadic target information together with the computed displacement of visual space during their eye movement to postdict the target position to pre-saccadic space, thereby generating a target representation that guides saccade targeting and influences the perception of space.

## Data Availability

The datasets created and analyzed in the current study are available from the OSF repository (https://osf.io/dtu4c/?view_only=58fdf3689ea94226bf39caf7c3ed2a88).
